# Burst-Like Subcutaneous Electrical Stimulation Induces BDNF-Mediated, Cyclotraxin B-Sensitive Central Sensitization in Rat Spinal Cord

**DOI:** 10.3389/fphar.2018.01143

**Published:** 2018-10-10

**Authors:** Jeffri Retamal, Andrea Reyes, Paulina Ramirez, David Bravo, Alejandro Hernandez, Teresa Pelissier, Luis Villanueva, Luis Constandil

**Affiliations:** ^1^Laboratory of Neurobiology, Department of Biology, Faculty of Chemistry and Biology, University of Santiago de Chile, Santiago, Chile; ^2^Center for the Development of Nanoscience and Nanotechnology (CEDENNA), Santiago, Chile; ^3^Program of Molecular and Clinical Pharmacology, Institute of Biomedical Sciences, Faculty of Medicine, University of Chile, Santiago, Chile; ^4^Centre de Psychiatrie et Neurosciences, INSERM UMR 894, Paris, France

**Keywords:** subcutaneous electrical stimulation, hyperalgesia, C-reflex, windup, brain-derived neurotrophic factor, cyclotraxin-B

## Abstract

Intrathecal administration of brain derived neurotrophic factor (BDNF) induces long-term potentiation (LTP) and generates long-lasting central sensitization in spinal cord thus mimicking chronic pain, but the relevance of these observations to chronic pain mechanisms is uncertain. Since C-fiber activation by a high-frequency subcutaneous electrical stimulation (SES) protocol causes spinal release of BDNF and induces spinal cord LTP, we propose that application of such protocol would be a sufficient condition for generating long-lasting BDNF-mediated central sensitization. Results showed that application of burst-like SES to rat toes produced (i) rapid induction of hyperalgesia that lasted for more than 3 weeks, (ii) early increase of C-reflex activity followed by increased wind-up scores lasting for more than 1 week, and (iii) early increase followed by late decrease in BDNF protein levels and phosphorylated TrkB that lasted for more than 1 week. These changes were prevented by the TrkB antagonist cyclotraxin-B administered shortly before SES, while hyperalgesia was reversed by cyclotraxin-B administered 3 days after SES. Results suggest that mechanisms underlying central sensitization first involve BDNF release of probably neuronal origin, followed by brief increased expression of likely glial BDNF and pTrkB that could switch early phase sensitization into late one.

## Introduction

Chronic pain is characterized by its persistence in time even when the primary cause had already been resolved. It is causally related to a phenomenon called central sensitization characterized by hyperalgesia, allodynia, and expansion of receptive field, which mainly results, among other factors, from long-lasting increases in efficacy of synaptic communication between nociceptive afferent fibers and some specific dorsal neurons, termed long-term potentiation (LTP) (Latremoliere and Woolf, 2009; Sandkühler, 2009). LTP can typically be induced in some synapses of the hippocampus (Bliss and Lømo, 1973), cerebral cortex (Artola and Singer, 1987) or spinal cord dorsal (Randic et al., 1993) horn by applying high-frequency electrical stimulation (e.g., 50–100 Hz) to corresponding presynaptic neurons. During persistent pain, LTP is assumed to be generated in dorsal horn synapses by a barrage of action potentials triggered from the injured peripheral tissue, which travels via nociceptive afferent C-fibers (Sandkühler, 2009). The increase in synaptic efficacy that defines the LTP process results from changes in phosphorylation and expression of some molecules in the spinal cord dorsal horn, as well as from the remodeling of specific neuronal circuits there (Latremoliere and Woolf, 2009; Sandkühler, 2009). In this context, inflammatory, neuropathic and other forms of chronic pain, are causally related to upregulation of a variety of pronociceptive mediators produced by neurons and glial cells in the spinal cord, i.e., glutamate (Kawamata and Omote, 1996; Andó and Sperlágh, 2013), ATP (Cui et al., 2014), cytokines (Whitehead et al., 2010), neurotrophins (Coull et al., 2005), which are accompanied by complex changes in dorsal horn expression of the corresponding receptors, i.e., glutamate, purinergic, cytokine, and BDNF receptors (Latremoliere and Woolf, 2009). In turn, intrathecal injection of these mediators to naïve animals can induce a behavioral pain response, but only the intrathecal injection of the neurotrophin BDNF and ATP has been found to produce a significant hyperalgesic status that is maintained for several weeks (Nakagawa et al., 2007; Constandil et al., 2011; Marcos et al., 2017).

The understanding of the specific role of each pronociceptive mediator in the initiation and maintenance of chronic pain is hindered by the fact that all the animal models used in chronic pain research produce variable degrees of peripheral tissue damage, which prevents the collection of reliable molecular, structural, functional, and behavioral information from the very beginning of pain elicitation, which is critical to differentiate the spinal mechanisms that initiate chronic pain (e.g., early LTP) from those involved in its maintenance (e.g., late LTP). Therefore, development of new experimental pain models of chronic pain devoid of peripheral lesion is a real need today. Here we propose the development of a new model of chronic pain in rat, devoid of peripheral lesion, based on the induction of spinal cord central sensitization by means of the application of a high-frequency subcutaneous electrical stimulation (SES) protocol to a rat hind paw, strong enough for activating nociceptive C-fibers, and to take advantage from this model to study the participation of BDNF in the initial stages of chronic pain. For this purpose, we conducted a long-term evaluation of the hyperalgesia developed in rats submitted to different burst-like SES protocols, as well as of the changes produced in nociceptive transmission properties in the spinal cord, using paw pressure testing and C-fiber-mediated reflex recording, respectively. By utilizing these experimental paradigms, we studied the effect of i.t. administration of the highly potent and selective allosteric TrkB antagonist cyclotraxin-B (CTX-B) (Cazorla et al., 2010), in order to give insights on the participation of the BDNF neurotrophin on the early and/or late neuroplastic mechanisms underlying spinal cord sensitization. Finally, the ability of burst-like SES to overexpress BDNF levels and to phosphorylate TrkB in lumbar spinal cord, as well as the susceptibility of these changes to CTX-B administration, were also studied using ELISA methods. The rats used were additionally studied regarding the potential capacity of high-frequency SES to produce peripheral lesion at the site of application, which is the main unwanted factor of experimental chronic pain models existing today during exploration of central sensitization mechanisms.

## Materials and Methods

### Animals

Adult male Sprague–Dawley rats (200–220 g) were purchased from the Animal Facility Centre of the Faculty of Medicine, University of Chile, and maintained in a controlled environment (12 h light/dark cycles and ambient temperature of 22°C) with food and water freely available. The animals were housed 3 to 4 per cage on a wood chip litter. All efforts were made to minimize the number of animals used. The experiments were performed in agreement with the Guide for the Care and Use of Laboratory Animals of NIH (National Research Council, 2011), Pain and Laboratory Animals: Publication Practices for Better Data Reproducibility and Better Animal Welfare (Carbone and Austin, 2016), and were approved by the local institutional Bioethics Committee of the University of Santiago of Chile (C-589). To perform the determinations required in the study, all the investigators were blinded to the experimental conditions. To determine the number of required rats, we use G^∗^Power 3 Software (Faul et al., 2007) for conducting sample size power analysis.

### High-Frequency Subcutaneous Electrical Stimulation (SES)

The rats were anesthetized with 2.5% isoflurane/97.5% O_2_ (Sigma, St. Louis, MO, United States) employing a latex diaphragm-modified rodent facemask, and stimulated electrically with high-frequency SES through a pair of steel electrodes subcutaneously placed in the fourth and fifth toes of the right hind paw. Three different high-frequency SES protocols were initially used: Protocol 1: 1-s pulse trains applied every 10 s during a 3-min period, each train composed by rectangular pulses of 1 ms and 7 mA intensity, at 100 Hz; this current intensity is slightly over the threshold current required for subcutaneous activation of C-fibers as calculated from the C-reflex study (see below); Protocol 2: similar to protocol 1, but applied during 20 min; Protocol 3: similar to protocol 1, but repeating the high-frequency SES after a 10-min interval. This last protocol produced reliable hyperalgesia beginning 1 h after SES application and lasting for more than 21 days, and was selected to be used for the remainder of the study (SES group). To explore the specificity of the hyperalgesic response to pulse frequency and current intensity used in protocol 3, two other pulse frequencies of 50 and 10 Hz were tested while maintaining the 7-mA current (50 Hz 7 mA and 10 Hz 7 mA groups); in addition, two other constant currents of 5 and 3 mA were tested but now maintaining the original 100 Hz frequency (100 Hz 5 mA and 100 Hz 3 mA groups). All the variants of the high-frequency SES utilized were generated with a S88 Grass stimulator, associated to a SIU5 Grass stimulus isolator unit and to a CCU1 Grass constant current unit (all from Astro-Med, Inc., West Warwick, RI, United States). Control rats were also anesthetized and the electrodes subcutaneously inserted into the toes, but no electrical stimulation was applied (SES-sham group). Once the different SES or SES-sham protocols were applied, the stimulating electrodes utilized for that purpose were removed.

### Mechanical Nociception Test

The paw pressure test was used to evaluate the threshold for mechanical nociception in SES-treated and SES-sham rats. The test consisted of the progressive application of a uniformly increasing pressure over the right hind paw using an Analgesy Meter (Ugo Basile, Italy), until a withdrawal reflex come on. To avoid injury, a cut-off value of 480 g was used. The mechanical nociception was evaluated before applying the high-frequency SES protocol, and 1 h and 1, 3, 7, 14, and 21 days post-SES protocol. Because induction of hyperalgesia in one paw sometimes leads to bilateral hyperalgesia, the mechanical thresholds of the paw ipsilateral and contralateral to the SES protocol were initially evaluated. Because no significant changes were found in the mechanical threshold in the contralateral leg, only the ipsilateral leg was tested for the remaining of the study.

### Protein Extraction and Quantification of BDNF and pTrk-B

L3–L5 spinal cord segments extracted were kept on ice, homogenized (Tissue Tearor model 985370, Biospec Products, Inc., United Kingdom) in lysis buffer (9803S, Cell Signaling Technology, Danvers, MA, United States) containing 1× sodium orthovanadate and 1× Roche complete protease inhibitor cocktail, and centrifuged at 7000 rpm (Hettich, universal model 320R, Tuttlingen, Germany) at 4°C for 15 min. The protein concentration was determined by the bicinchoninic acid method (BCA) using the kit BCA Protein Assay through a colorimetric reaction quantified in an FC multiskan ELISA reader (Thermo Fisher Scientific, Barrington, IL, United States). BSA curve was used as a standard to quantify the total protein concentration. Measurements were made at an absorbance of 562 nm.

Quantification of BDNF and pTrk-B was performed by ELISA according to manufacturer specifications. BDNF protein was quantified using the ELISA E_max_ Immunoassay System (Promega, Madison, WI, United States), while for quantifying pTrkB the ELISA test PathScan^®^ Phospho-TrkB (panTyr) (Cell Signaling Technology, Danvers, MA, United States) was used. In our experience, BDNF concentrations determined with ELISA are in a similar range to those detected by Western blot method.

### C-Reflex and Wind-Up Recording

The electromyographic (EMG) activity associated to the hind limb-flexion nociceptive reflex evoked by activation of C fibers of the sural nerve (C-reflex) was studied by using the method described by Falinower et al. (1994). All the procedures were performed on rats anesthetized with 0.8% isoflurane/99.2% O_2_ and placed on a homoeothermic blanket system (37 ± 0.5°C). Briefly, a pair of uncoated platinum low profile needle electrodes was inserted subcutaneously into the lateral part of the third and fourth toes. The electrical stimuli, consisting of single rectangular shocks of 1-ms duration with 2-time the current intensity required to elicit a threshold C-reflex, were delivered at 0.1 Hz from a Grass stimulator S11 associated with a SIU5 Grass stimulus isolator unit and to a CCU1 Grass constant current unit. The EMG responses were recorded via another pair of platinum electrodes inserted through the skin into the ipsilateral biceps femoris muscle. The responses were amplified with a Grass P511 preamplifier (Astro-Med, Inc., West Warwick, RI, United States), and digitized at 4 KHz and integrated into a 150–450 ms post-stimulus time window using a PowerLab 2/20 (ADInstruments, Castle Hill, NSW, Australia) connected to a IMAC computer (Apple INC, CA, United States). Plotting integrated C-reflex responses versus increasing stimulus intensities allowed detecting the C-fiber threshold current intensity in naïve rats (6.5 ± 0.3 mA) by linear regression procedure. C-reflex data was recorded as averages of 15 consecutive windows of integrated C-reflex (stimulation at 0.1 Hz, 2 × threshold current intensity). To study the potentiation of C-reflexes to repetitive stimulation, known as wind-up, the stimulating frequency was incremented to 1 Hz while maintaining the 2 × threshold current intensity. The wind-up was evaluated by using data only from the first seven consecutive windows, which show incremental trend, and calculated as the slope of the regression line fitted to these seven-point data. CTX-B or saline was i.t. injected prior to applying the high-frequency SES or the sham protocol and the C-reflex and wind-up followed for 30, 60, 90, and 120 min. Afterward, the stimulating and recording electrodes were removed and the rat returned to its cage. Seven days after, C-reflex and wind-up studies were repeated on the same animals, and thereafter the rats were sacrificed by means of an overdose of isoflurane.

### Inhibition of TrkB Receptor

CTX-B, manufactured as Tat-cyclotraxin-B (Bio S & T, Montreal, QC, Canada), was dissolved in saline and administered intrathecally as a single dose of 40 μg/kg, in a 10 μl volume. CTX-B was administered preventively, 90 min before the application of the high-frequency SES protocol (*n* = 6), and curatively 3 days after application of high-frequency SES (*n* = 6), according to Constandil et al. (2012). Both groups were evaluated daily for 7 days. On day 7, after the last algesimetric measurement, the animals were sacrificed by an overdose of anesthesia and the lumbar spinal cord (L3–L5 segments) was removed for proteins analysis.

### Evaluation of Safety of the High-Frequency SES Protocols in Animals

To study whether the SES protocols produce or not inflammation in the rat’s right hind paw, increase in volume, flushing, temperature were determined in the hind paw prior and 10 and 60 min after the application of the high-frequency SES protocols, by means of Vernier caliper, visual inspection and a contact thermistor thermometer applied on the skin surface of the paw, respectively. In addition, plasma extravasation of Evans blue dye in the stimulated hind paw was also determined using a modification of the technique described by Gonzalez et al. (2005). Briefly, 50 mg/kg of Evans blue dye was injected into a tail vein of the rat 10 min before application of SES to the hind paw, and serial images of the paw were recorded with a Sony WX500 digital camera prior and 10 and 60 min after the application of the high-frequency SES protocol. Extravasated dye was evaluated through change in light reflectance (8 bit 0–255 scale) by measuring the mean value of pixels included in a 5 mm × 5 mm square region of the dorsomedial part of the stimulated hind paw, by using the Fiji image processing package (Laboratory for Optical and Computational Instrumentation, University of Wisconsin–Madison, Madison, WI, United States).

### Data Analysis

The results were expressed as means ± SEM and analyzed with the statistical software GraphPad Prism 6.0 (GraphPad Software Inc., San Diego, CA, United States). To assess the time-course of changes induced by the high-frequency SES protocol, analyses were performed by comparing pre and post-treatment scores using the Kruskal–Wallis test followed by the *post hoc* Dunn’s multiple comparisons test. Non-parametric statistics was chosen because animal groups were composed by six rats, a sample size that usually not allows to demonstrate a Gaussian distribution of data group. The GraphPad Prism 6.0 was used to calculate area under curves and slopes of linear regression fitting in the wind-up study. In all comparisons, statistical significance was established at *p* < 0.05.

## Results

### High-Frequency SES Induced Long-Lasting Hyperalgesia in the Rat

**FIGURE 1:**
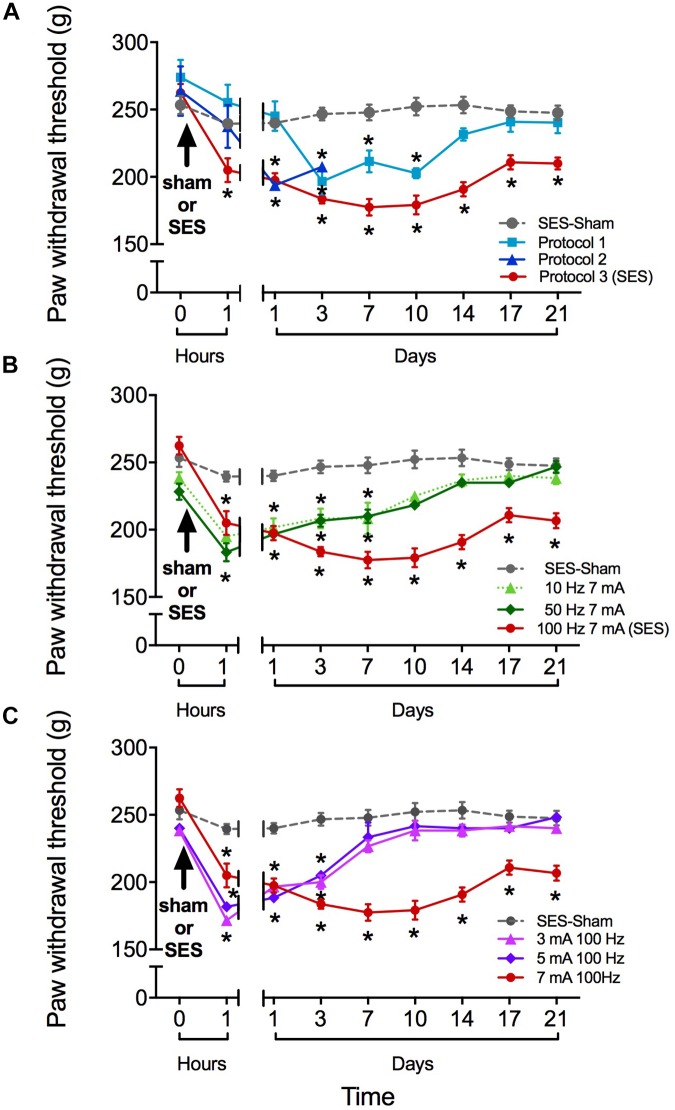
Paw pressure threshold of rats submitted to different high-frequency SES protocols. **(A)** SES-sham protocol and three high frequency protocols. Protocol 1: 100 Hz, 7 mA, 3 min; Protocol 2: 100 Hz, 7 mA, 20 min; Protocol 3 (SES): 100 Hz, 7 mA, 3 min^∗^2. **(B)** SES-sham protocol and 10 Hz, 7 mA; 100 Hz, 50 Hz, 7 mA and 7 mA (SES). **(C)** SES-sham protocol and 3 mA, 100 Hz; 5 mA, 100 Hz and 7 mA, 100 Hz (SES). Values are mean ± SEM (*n* = 6 rats per group). Asterisks indicate a significant intragroup difference (^∗^*p* < 0.05) when post-SES values were compared with the pre-SES score at time zero (Kruskal–Wallis test followed by the *post hoc* Dunn’s multiple comparisons test).

As shown in **Figure 1**, the SES-sham group exhibited no significant change in the paw pressure threshold during the 21 days of the study, indicating that the mere subcutaneous insertion of the stimulating electrodes under the skin of toes does not significantly modify the nociceptive threshold to subsequent mechanical nociceptive testing of rats. In contrast, all groups receiving high frequency SES showed a significant decrease of the paw pressure threshold during variable time intervals, depending on the protocol used. In the group receiving the SES protocol 1 (3-min stimulation, 7 mA, 100 Hz), the paw pressure threshold decreased on day 3 post-stimulation and remained significantly decreased until day 10, as compared to the basal value obtained prior to electrical stimulation (**Figure 1A**). In the group receiving the SES protocol 2 (20-min stimulation, 7 mA, 100 Hz), the paw pressure threshold remained significantly decreased since day 1 post-stimulation until day 3, compared to the basal value (**Figure 1A**); the experiment was stopped on day 3 and the rats (*n* = 2) euthanized because the paw showed an electrical burn injury. In the group stimulated with protocol 3 (2 × 3-min stimulation, 7 mA, 100 Hz), the paw pressure threshold decreased since 1 h post-SES until to the end of the experiment on day 21 post-SES (**Figure 1A**). Animals subjected to this protocol (SES group) showed no visible injury in the area stimulated. Furthermore, none of the signs associated with inflammation (increase in volume, flushing, increased temperature, and plasma extravasation) were observed in the hind paw of these animals 10 min or 1 h post-SES (**Table 1**). However, we cannot totally rule out that there has been some subtle alteration in the stimulated paw of the SES group that was not detected by our inflammatory assays.

**Table 1:** Inflammatory parameters measured in animals submitted to the high-frequency SES protocol.

Stimulated hind paw	Previous SES	Ten minutes post-SES	One hour post-SES
Temperature (°C)	31.1 ± 0.8	31.31 ± 0.6	31.5 ± 0.5
Skin color	Normal	Normal	Normal
Hind paw thickness (mm)	4.9 ± 1.0	4.8 ± 2.1	4.8 ± 2.6
Plasma extravasation	83.7 ± 6.3	92.1 ± 8.4	87.9 ± 8.3

*Values are mean ± SEM (n = 6 rats per group). No significant differences between previous basal and 10 min or 1 h post-SES values were found (Kruskal–Wallis test).*

Reduction in SES frequency to 50 or 10 Hz (but maintaining the 7-mA current intensity) resulted in a less lasting effect of SES, since in these conditions the decrease in mechanical nociceptive threshold only lasted for 7 days (**Figure 1B**). Alternatively, reduction of SES current intensity to 5 or 3 mA (but maintaining the 100-Hz frequency of stimulation) led to a decrease of the mechanical nociceptive threshold that lasted for only 3 days (**Figure 1C**).

### High-Frequency SES Induced Biphasic Effects in BDNF Expression and TrkB Phosphorylation in Rat Lumbar Spinal Cord

**FIGURE 2:**
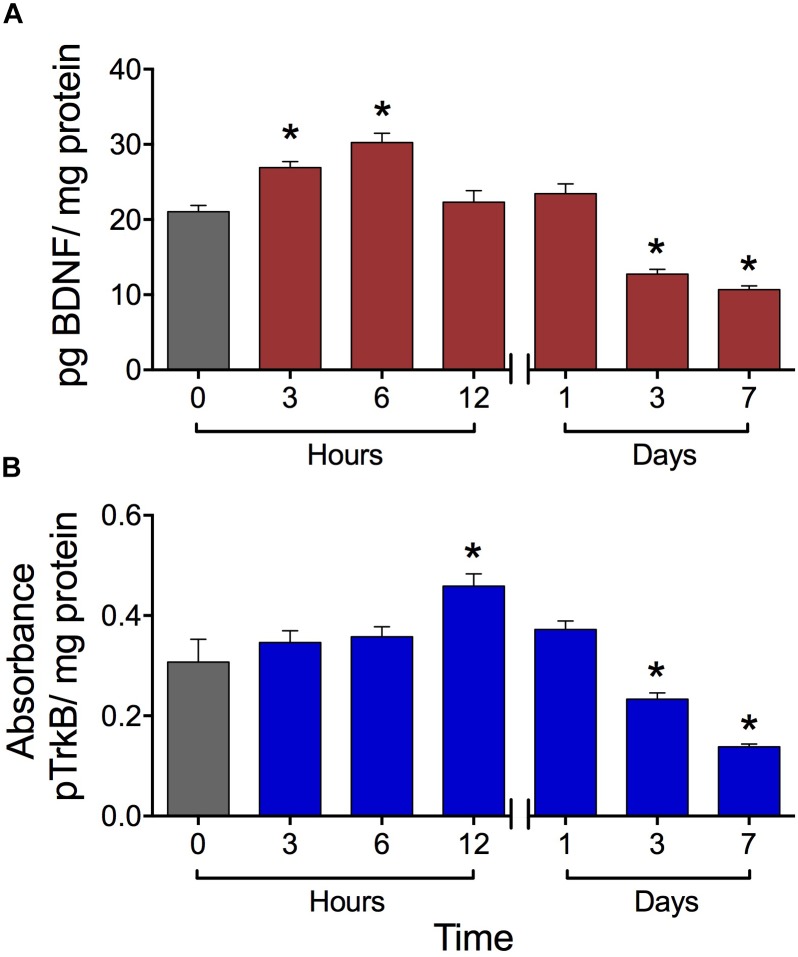
Expression levels of BDNF **(A)** and pTrkB **(B)** in the L3–L5 laminae of dorsal lumbar spinal cord of rats submitted to the high-frequency SES protocol. Values are means ± SEM (*n* = 6 rats per group). BDNF and pTrkB were measured with ELISA test. The BDNF was expressed as pg BDNF/mg protein due to the absorbance was compared with a concentration reference curve, while that for pTrkB we have a not a reference curve. Asterisks show a significant difference (^∗^*p* < 0.05) respect to time zero (black bar) (Kruskal–Wallis test followed by the *post hoc* Dunn’s multiple comparisons test).

The high-frequency SES applied to the hind paw modified in a biphasic manner the BDNF concentration in the lumbar spinal cord. Initially, at 6 h post-stimulation, the BDNF expression level increased by 42% with respect to the basal level. At 12 and 24 h post-SES the BDNF levels were similar to the basal one, whereas at days 3 and 7 post-stimulation the BDNF expression levels were significantly lower (around 50% decrease) than the basal level (**Figure 2A**). The concentration of pTrkB in lumbar segments of the spinal cord also presented a biphasic variation, but with a phase shift of the peak of 6 h. Indeed, pTrkB levels were significantly higher 12 h after SES stimuli, while showing a significant decrease 3 and 7 days after the electrical stimuli compared to the basal measure prior to SES (**Figure 2B**).

### Intrathecal Cyclotraxin-B Prevented and Reversed Mechanical Hyperalgesia and the Late Decrease in Lumbar Spinal Cord Expression Levels of BDNF and pTrkB Induced by High-Frequency SES

**FIGURE 3:**
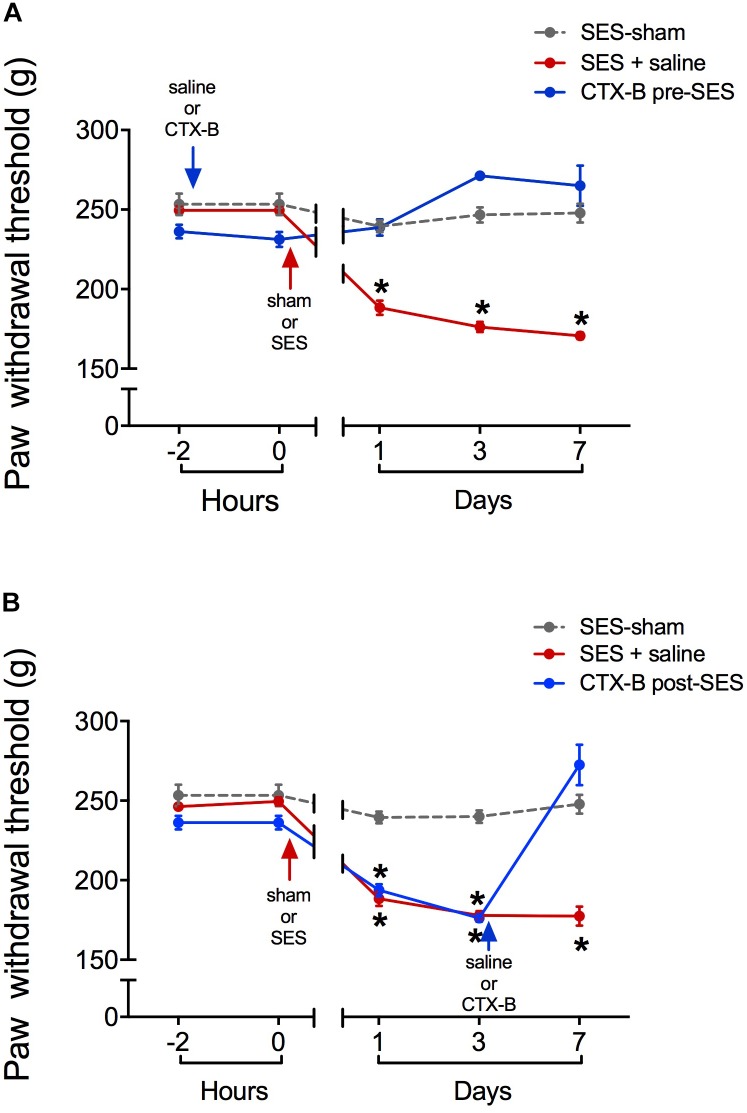
Antagonistic effects of CTX-B upon high-frequency SES induced-hyperalgesia, as revealed by paw pressure thresholds. **(A)** CTX-B or saline was administered 90 min before SES (CTX-B SES-sham, CTX-B pre-SES), **(B)** CTX-B or saline was administered 3 days after SES (CTX-B SES-sham and CTX-B post-SES). Values are means ± SEM (*n* = 6 rats per group). Asterisks indicate a significant intragroup difference (^∗^*p* < 0.05) when post-SES scores were compared with the pre-SES score (time 0) (Kruskal–Wallis test followed by the *post hoc* Dunn’s multiple comparisons test).

**FIGURE 4:**
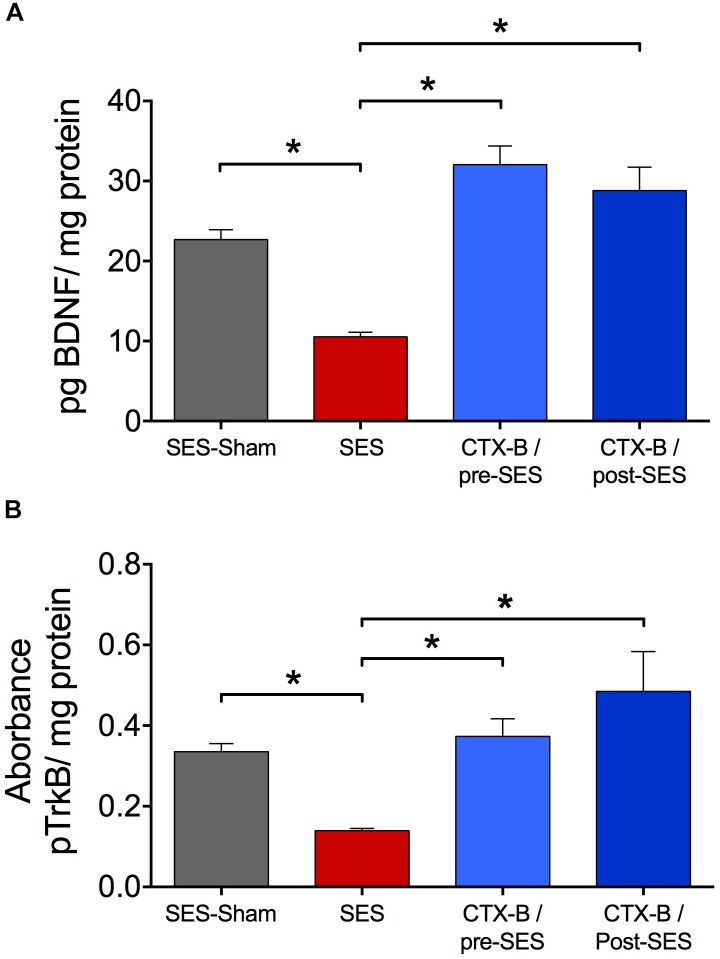
Antagonistic effects of CTX-B upon high-frequency SES induced-decreased in the expression levels of both BDNF **(A)** and pTrkB **(B)** on day 7 after SES. Values are means ± SEM (*n* = 6 rats per group). Asterisks indicate a significant intergroup difference (^∗^*p* < 0.05, Kruskal–Wallis test followed by the *post hoc* Dunn’s multiple comparisons test).

CTX-B, administered i.t. 90 min before SES, prevented the decrease in paw pressure threshold produced by the SES while saline did not (**Figure 3A**). In agreement, in animals receiving pre-SES injection of CTX-B, the expression levels of BDNF (**Figure 4A**) and pTrk-B (**Figure 4B**) found in lumbar spinal segments on day 7 post-SES were not significantly different to those found in SES-sham animals. CTX-B administered i.t. 3 days post-SES significantly reverted the decrease in paw pressure threshold produced by the high-frequency SES protocol, the paw withdrawal threshold on day 7 being returned to the pre-SES score (**Figure 3B**). Intrathecal CTX-B, given on day 3 post-SES, prevented the late decrease in BDNF and pTrk-B expressions, as on day 7 post-SES these animals did not show significant differences in expression levels of the transcripts with respect to corresponding levels found in the SES-sham group (**Figures 4A,B**, respectively).

### High-Frequency SES Induced Differential Short- and Long-Term Increments of Nociceptive Transmission in Rat Spinal Cord: Preventive Effect of CTX-B

**FIGURE 5:**
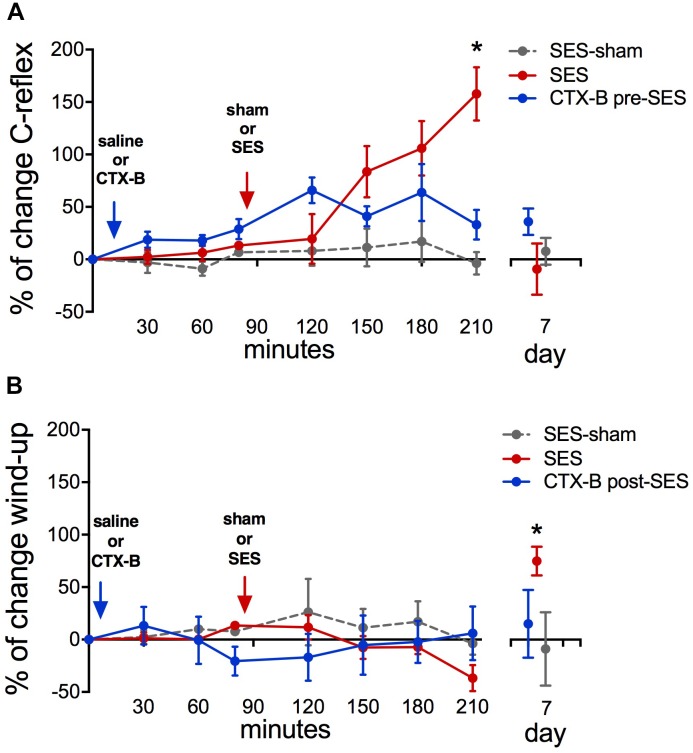
Percent of change in C-reflex activity **(A)** and wind-up score **(B)** after applying the high-frequency SES protocol and the effects of CTX-B administered 90 min before SES. Values are means ± SEM (*n* = 6 rats per group). Asterisks indicate a significant intragroup difference when post-SES scores were compared with the pre-SES value (^∗^*p* < 0.05, Kruskal–Wallis test followed by the *post hoc* Dunn’s multiple comparisons test).

Animals subjected to the high-frequency SES protocol showed increased integrated C-reflex activity 60 min following SES, as related to the pre-SES baseline value, which attained statistical significance upon 120 min post-SES (**Figure 5A**). In these animals, the integrated C-reflex responses returned to basal values 7 days after application of the SES protocol. No significant changes were observed in the time-course of the C-reflex response in animals subjected to the SES-sham protocol (**Figure 5A**). Since C-reflex responses, unlike mechanical hyperalgesia, returned spontaneously to basal values 7 days after application of the SES protocol, the antagonistic effect of CTX-B administered post-SES on the integrated C-reflex was not tested.

Wind-up scores did not change along the 210-min period of recording after applying the high-frequency SES protocol, as related to baseline scores obtained prior to SES application. However, on day 7 after SES, wind-up activity was significantly increased with respect to pre-SES scores (**Figure 5B**), which is indicative of a late neuroplastic event. No significant changes were observed throughout the experiment in C-reflex wind-up scores from animals subjected to the sham-SES protocol (**Figure 5B**).

Administration of CTX-B 90 min before the application of the high-frequency SES protocol prevented the increase in C-reflex activity elicited by SES (**Figure 5A**). CTX-B administered prior to SES also prevented the late increase of wind-up scores (**Figure 5B**).

## Discussion

We found that application of a high-frequency SES protocol, strong enough to activate C-fibers in the nociceptive afferent pathway, is a sufficient condition for generating long-lasting central sensitization in naïve animals, as revealed by (i) rapid induction of hyperalgesia that lasted for more than 3 weeks, (ii) early increase of integrated C-reflex activity followed by increased wind-up scores lasting for more than 1 week, and (iii) early increase in the levels of BDNF protein and phosphorylated TrkB, followed by late decreased levels of both molecular markers that lasted for more than 1 week. All these changes were prevented by intraperitoneal administration of CTX-B before SES, and intrathecal injection of CTX-B 3 days after SES also was effective to reverse the hyperalgesia induced. The SES protocol used did not generate noticeable injury in peripheral tissue, as revealed by unchanged values either in volume, flushing, temperature, or plasma extravasation of the stimulated hind paw.

Nociceptive sensitization induced by peripheral electric stimulation was first demonstrated by Woolf and Wall (1986), who showed that low frequency (2 Hz) conditioning electrical stimuli at an intensity sufficient to activate C-afferent fibers result in a short-term (3 h) heterosynaptic facilitation of the flexor motoneuronal response in anesthetized decerebrate spinal rat. Later, other authors showed that low-frequency electric stimulation (10 Hz) of peripheral C-fibers evoked a longer (48 h) but still short, central sensitization to mechanical stimuli in awake behaving rats (Hathway et al., 2009). Thus, low-frequency stimulation of peripheral C-fibers does not mimic the temporal profile of pain observed in most animal models of chronic pain, which persists quite stable for more than 15 days. Here, we showed that a 3-min series of high-frequency electric stimulation at 100 Hz, applied at 7 mA to the rat hind paw, which is slightly over the threshold current required for subcutaneous activation of C-fibers, produced heterotopic mechanical hyperalgesia that persisted for 1 week. Moreover, upon repetition of the series 10 min after, the hyperalgesic status was prolonged for at least 3 weeks. Such an enduring hyperalgesia is quite similar to that found in preclinical models of chronic pain, at least in terms of time-course and stability of the central sensitization process. In humans, repeated electrical stimulation of the skin with trains of 100 Hz at 10–20 × individual detection threshold, which supposedly targeted peptidergic C-fibers, produced a gradual increase in both the homotopic and heterotopic pain, which persisted for about 200 min (Klein et al., 2004), thus providing a link between the present results and clinical observations concerning the hyperalgesic effect of high-frequency stimulation protocols. Central sensitization can also be triggered by natural stimuli applied to the skin such as mechanical, thermal, or by irritating reactive chemicals, but also by a variety of mediators released in injured tissue, such as ATP, cytokines, and neurotrophins (Sandkühler, 2009). However, the major advantages of electrical stimulation over any other types of stimulation are brevity and strict control in time of stimulus onset, cut-off, frequency, pulse pattern, and duty cycle, as well as the absence of peripheral injury and associated peripheral sensitization phenomena. It is worthy to note that in spite of no plasma extravasation and edema were observed in the stimulated hind paw during the course of the present experiments, there is older data showing that antidromically activated C afferents could lead to peripheral release of neuropeptides that could result in vasodilation and plasma extravasation in the skin (Gee et al., 1997; Hathway et al., 2009), which are key components of neurogenic inflammation. Nevertheless, it has been reported that unlike neurogenic inflammation produced by capsaicin, mustard oil or histamine, antidromic electrical stimulation of C-fibers did not evoke spontaneous activity in the polymodal fibers tested nor did it markedly affect their mechanical and thermal excitability in periods of up to 1 h after stimulation (Reeh et al., 1986), thereby indicating absence of peripheral sensitization. Further, the literature shows that the time-course of symptoms (vasodilation, plasma extravasation, or edema) for the electrically evoked, C-fiber mediated neurogenic inflammation did not last for more than 1 h (Gee et al., 1997; Nakagawa et al., 2007; Hathway et al., 2009) and, therefore, they can be ruled out as factors involved in the more lasting central effects produced by the SES protocol.

Ying et al. (2006) generated bilateral mechanical allodynia in the rat hind paw by using a pair of stainless-steel hooks for direct electrical stimulation of the exposed sciatic nerve with 30 Volt pulses (100 Hz, 20 trains of 2 s duration at 10 s intervals). Without proper control of the stimulating current intensity, this procedure may provide a very strong current that will flow through across the entire nerve and, as discussed by Ying et al. (2006), a slight degeneration of some myelinated and unmyelinated fibers in the sciatic nerve was observed under an electronic microscope which might lead to activate peri-sciatic immune cells. As reported elsewhere, bilateral allodynia can be created in rats by microinjection of immune activators around one healthy sciatic nerve, where intense immune activation produces bilateral allodynia, while low level immune activation produces allodynia only in the microinjected side (Milligan et al., 2003). In some initial experiments, we also included testing of the mechanical threshold of the hind paw contralateral to the applied SES. Unlike the ipsilateral hind paw, no hyperalgesia could be demonstrated in the contralateral one (results not shown).

Central sensitization in the spinal cord has been linked to immediate-onset, transcription-independent, NMDA receptor-mediated mechanisms, such as LTP (Sandkühler, 2009), and afterward maintained by a late-onset, transcription-dependent mechanism that include ERK signaling to the nucleus and subsequent phosphorylation of CREB, with increased expression of early genes codifying for c-fos, COX_2_ and the neurotrophin BDNF, as well as for other late response genes (Latremoliere and Woolf, 2009). In the spinal cord, early-phase LTP could be rapidly induced (within minutes) as an activity-dependent regulated process virtually by any type of high-frequency burst-like stimulation at C-fiber intensity, without requiring of protein synthesis (Sandkühler, 2009). The reduced paw pressure threshold and the increased C-reflex activity detected 1 to 2 h following SES, short before BDNF and pTrkB upregulation, are most likely the behavioral consequence of the development of an early-onset, activity-dependent, LTP-based, central sensitization process. Furthermore, since the changes induced by SES in paw pressure threshold and C-reflex activity were prevented by administration of CTX-B, a drug that is unable to modify the nociceptive thresholds in normal non-sensitized mice (Constandil et al., 2012), it is likely that the whole process was triggered by the release of BDNF from primary afferents due to SES. This interpretation is supported by the following observations: (i) high-frequency SES, but not low-frequency SES, of afferent sensory nerves at C-fiber intensity causes spinal release of BDNF (Lever et al., 2001); (ii) intrathecal injection of BDNF produces CTX-B-sensitive enduring mechanical hyperalgesia in rats, which imitates that induced by high-frequency SES (M’Dahoma et al., 2015); (iii) full-length TrkB receptors were present at somato-dendritic membranes of lamina II neurons in the rat and mouse dorsal horn (Salio et al., 2005); (iv) binding of BDNF to the TrkB receptor regulates neural response and synaptic function in the dorsal horn output neurons through a variety of neuroplasticity mechanisms, including increased phosphorylation of NMDA receptor subunits NR1 (Slack et al., 2004; Liu et al., 2015) and NR2B (Peng et al., 2012; Ding et al., 2015; Li et al., 2017); (v) activation of NMDA receptors downstream to TrkB signaling is essential for behavioral expression of the mechanical hyperalgesia induced by intrathecal BDNF (Marcos et al., 2017). It is then conceivable that BDNF released by C-fibers during SES application rapidly facilitates the induction of NMDA-dependent early-phase LTP in the spinal cord thereby leading to central sensitization, as reflected by the lower paw pressure threshold and the increased C-reflex activity. This effect in spinal cord parallels BDNF-dependent postsynaptic LTP induced in the hippocampus either by long-lasting high-frequency (Chen et al., 1999) or by repeated theta burst (Kang et al., 1997) presynaptic stimulation, but unlike spinal cord, in the hippocampus the origin of BDNF is most likely of postsynaptic origin (Edelmann et al., 2015).

Unlike paw pressure threshold and C-reflex activity, spinal wind-up was not modified soon after the administration of SES, perhaps because BDNF-mediated phosphorylation of NMDA receptors in the postsynaptic density did not provide the extra-calcium current required to significantly increase wind-up. Alternatively, failure of SES to rapidly potentiate wind-up could be intrinsic to the experimental design, since wind-up is a form of reversible homosynaptic potentiation, i.e., the synapses activated by the conditioning and test inputs should be the same (Jeftinija and Urban, 1994). In fact, because the high frequency SES (the conditioning stimulus) was applied with electrodes placed in the fourth and fifth toes while wind-up (the tested response) was induced by low-frequency stimulation of the third and fourth toes, it is likely that the SES-activated synapses (and the corresponding phosphorylated NMDA receptors by BDNF release) were different to those tested for wind-up responsiveness. Nevertheless, once late, transcription-dependent plasticity has likely been developed, which entails widespread trafficking of new proteins to specific targets in the whole neuron, thereby including synapses to be tested for wind-up, the wind-up scores increased (e.g., 1 week after SES stimulation), as occurs in monoarthritis (Constandil et al., 2009) and neuropathic (D’Mello et al., 2011) chronic pain models.

High-frequency SES increased the expression levels of BDNF protein and pTrkB in lumbar spinal cord tissue, an effect that lasted around 6 to 12 h. Previous work demonstrated that sciatic nerve electrical stimulation (20 Hz at C-fiber strength) led to increased BDNF mRNA and TrkB mRNA expressions in rat dorsal root ganglion and dorsal horn, respectively, 2 h after stimulation (Mannion et al., 1999). Because these changes in expression of BDNF and TrkB transcripts occurred a few hours after the triggering of the hyperalgesia, these signal pathways appear relevant to the maintenance of central sensitization rather than its induction. The higher expression of BDNF protein observed 3–6 h after applying the SES protocol probably represents induction of BDNF in microglia, as indeed occurs in neuropathic pain models (Coull et al., 2005). Actually, in the rat and mouse dorsal horn the neuronal BDNF expression is restricted to primary afferent terminals which occurs mainly in laminae I and II, where BDNF is stored in synaptic vesicles (Salio et al., 2005; Merighi et al., 2008), and therefore the large bulk of BDNF protein we measured in the dorsal horn likely correspond to microglial BDNF transcripts. This rather late production of BDNF in spinal cord microglia could result from microglial activators, such as ATP and fractalkine (CX3CL1), which are released from stimulated primary afferent neurons in naïve and hyperalgesic animals (for review see Taves et al., 2013) and activate microglia via the corresponding P2X, TrkB, and CX3CR1 receptors and the associated downstream p38 MAPK pathway (Gong et al., 2009; Trang et al., 2009; Zhuang et al., 2007). It has been shown that microglial derived BDNF contributes to pain hypersensitivity through phosphorylation of the NMDA receptor subunits NR1 via ERK and PKC (Slack et al., 2004) and NR2B via Fyn kinase (Li et al., 2017), and also through the disinhibition of nociceptive processing in the dorsal horn via downregulation of the K^+^-Cl^−^ cotransporter KCC2 (Ferrini and De Koninck, 2013). Finally, BDNF also triggers long-term neuronal plasticity in the brain, and possibly in the spinal cord, via the activation of TrkB-ERK-CREB cascade, thereby signaling for a multitude of proteins involved in protein synthesis-dependent late-phase LTP (Minichiello, 2009). Thus, the activity-dependent increases of spinal BDNF and TrkB are temporally and functionally well positioned to switch early-phase LTP in the spinal cord into late-phase LTP.

Of note, 3 and 7 days after SES application, the expression levels of both BDNF protein and pTrkB in lumbar spinal cord tissue were found to be decreased below the pre-SES levels, as measured by ELISA method. Unexpectedly, i.t. CTX-B administered 3 days after SES application, normalized the decreased expression of both transcripts, when tested by ELISA on the day 7 post-SES. This suggests that decreased BDNF and pTrkB expressions are the result of an active BDNF-mediated, negative feedback process, which can be removed by CTX-B. In this regard, BDNF-stimulated internalization of TrkB receptors via rapid ubiquitination and degradation has been recently shown (Proenca et al., 2016), but BDNF-mediated BDNF autoregulation has never been reported. Instead, BDNF-positive feedback loops mediating transcriptional BDNF upregulation in cortical neurons (Tuvikene et al., 2016) and in microglia (Zhang et al., 2014) have been proposed. Also intriguing is that CTX-B administered 3 days after SES, reverted the already established sensitization process despite the fact that at this time-point the spinal cord BDNF and pTrkB levels were lower than the baseline pre-SES levels. This argues for two complementary hypotheses: (i) once created, the central sensitization process is maintained by a BDNF-dependent mechanism, likely of microglial origin, and (ii) the reduced BDNF/TrkB signaling observed 3 days after SES is sufficient for maintaining the already installed sensitization process. Support for these assumptions could be found in the observation that during chronic pain BDNF can overexpress NR2B-containing spinal NMDA receptors either via non-genomic SHP2/PSD-95/NR2B signaling (Ding et al., 2015) or by promoting CREB-mediated transcription of NR2B subunit (Liang et al., 2016). In this context, it is conceivable that the normal (or even reduced) BDNF/TrkB signaling in spinal cord, which usually do not correlate with hyperalgesia in healthy animals, in sensitized ones could lead to significant phosphorylation of the overexpressed NR2B-containing NMDA receptor population, thereby contributing to the maintenance of the sensitization process. If so, the concomitant hyperalgesia should be sensitive to TrkB receptor blockade by CTX-B, in spite of the reduced level of BDNF/TrkB signaling at this time-point. An alternative, but not necessarily contradictory, explanation is that the relevant factor for BDNF-induced neuroplastic effects in spinal cord (like LTP or spinal sensitization) is not the level of BDNF/pTrkB-like immunoreactivity, as assessed by ELISA or Western blotting, but the amount of microglial released BDNF that may bound TrkB receptors. Unfortunately, detection of released BDNF in micro dialysates from animal brains has proven to be extremely difficult because of its low concentration, i.e., at the sub-nanogram/ml level (however, see Yoo et al., 2016).

Finally, we cannot discard that some degree of sensitization in primary afferents could be due to unknown modifications induced by the SES protocol within the DRG. Indeed, electrical activation of A delta and C-fibers has been shown to increase pERK in dorsal root ganglion neurons (Dai et al., 2002). However, whether enhanced ERK signaling in nociceptive afferents could be involved in the triggering of peripheral mechanical hyperalgesia/allodynia remains unexplored, in part because the mechanosensitive channel (or channels) that mediate mechanical pain has not been identified.

## Conclusion

In summary, our findings demonstrate that a high-frequency SES protocol at intensity slightly over the C-fiber threshold is a sufficient condition for generating rapid and long-lasting sensitization of rat dorsal horn neurons that resembles chronic pain features, as assessed by either phasic mechanical or phasic/repetitive electric nociceptive stimuli that targeted C-fibers. The SES protocol used herein allowed for clear-cut dissection of early and late events underlying central sensitization in the spinal cord, because it admits immediate assessment of nociception and does not generate any noticeable injury in peripheral tissue that may constitute a confounding factor during nociceptive evaluation. Using CTX-B, a slowly reversible TrkB antagonist, we determined that the mechanisms underlying early sensitization are primarily mediated by activity and involve release of BDNF of probably neuronal origin, shortly followed by brief increased expression of likely glial BDNF that can switch early-phase sensitization into late one, thus substantiating CTX-B as a potential powerful tool for novel therapeutic strategies aimed to prevent or even treat some forms of chronic pain.

## Author Contributions

JR and LC provided ideas or concepts for definition of intellectual context, particularly designed and performed the experiments. JR, AR, PR, and TP performed the research. JR, PR, and DB contributed new reagents and analytic tools. JR, AH, TP, LV, and LC analyzed the data. AH and LC wrote the paper. All authors read and approved the final version of the manuscript.

## Conflict of Interest Statement

The authors declare that the research was conducted in the absence of any commercial or financial relationships that could be construed as a potential conflict of interest.
